# The variation of coverage and access to palliative care for cancer patients in eight European countries: an exploratory vignette approach

**DOI:** 10.1186/s12904-025-01831-1

**Published:** 2025-07-07

**Authors:** Nathan Shuftan, Mark Dayan, Sarah Scobie, Bee Wee, Antoniya Dimova, Elka Atanasova, Liubove Murauskiene, Coralie Gandré, Zeynep Or, Gonçalo Figueiredo Augusto, Triin Habicht, Kristina Köhler, Kristiina Kahur, Bertil Axelsson, Madelon Kroneman, Judith de Jong, Anke de Veer, Wanda Bemelmans, Chantal Pereira, Ajay Aggarwal, Joanna Davies, Ewout van Ginneken, Dimitra Panteli

**Affiliations:** 1 Department of Healthcare Management, Technische Universität Berlin, H 80, Straße Des 17. Juni 135, Berlin, 10623 Germany; 2 Nuffield Trust, 59 New Cavendish Street, London, W1G 7LP UK; 3 Nuffield Department of Medicine, University of Oxford, Old Road Campus, Oxford, OX3 7BN UK; 4 Medical University of Varna, 55 Marin Drinov Str, Varna, 9002 Bulgaria; 5 Department of Public Health, Faculty of Medicine, Institute of Health Sciences, Vilnius University, M. K. Čiurlionio G. 21/27, Vilnius, 03101 Lithuania; 6 Institute for Research and Information in Health Economics (IRDES), 21-23, Rue Des Ardennes, Paris, 75019 France; 7 NOVA National School of Public Health, Public Health Research Centre, Comprehensive Health Research Center, CHRC, NOVA University Lisbon, Rua da Junqueira, 100, Lisbon, 1349-008 Portugal; 8 WHO Barcelona Office for Health Systems Financing, Sant Pau Art Nouveau Site (La Mercè Pavilion), Sant Antoni Maria Claret, 167, Barcelona, 08025 Spain; 9 WHO Country Office, Paldiski Road 81, Tallinn, 10617 Estonia; 10 HC Management Consulting Ltd, Tallinn, Estonia; 11 Department of Radiation Sciences, University Hospital of Umeå, Daniel Naezéns Väg, Umeå, 907 37 Sweden; 12 Nivel, Netherlands Institute for Health Services Research, Otterstraat 118, Utrecht, CR 3513 The Netherlands; 13 Netherlands Comprehensive Care Organisation (IKNL), Godebaldkwartier 419, Utrecht, DT 3511 The Netherlands; 14 King’s College London, Guy’s Campus, Great Maze Pond, London, SE1 1UL UK; 15 George’s University NHS Foundation Trust, Blackshaw Road, London, SW17 0QT UK; 16 European Observatory On Health Systems and Policies, WHO European Centre for Health Policy, Eurostation (Office 07C020), Place Victor Horta/Victor Hortaplein, 40/10, 1060 Brussels, Belgium

**Keywords:** Palliative care, Coverage, Access, Cost-sharing, Benefit package, Financial protection

## Abstract

**Background:**

Palliative care aims to maintain quality of life and offer treatment and person-centred care options for people with serious end-stage illnesses and their families. The purpose of this exploratory study was to compare the statutory coverage and access to palliative care for adult services for people with cancer in 8 European countries using a vignette approach.

**Methods:**

We used a patient vignette to examine coverage and access to palliative care services across Europe. The palliative care vignette describes a pathway based on guidance for best practices of palliative care patients with incurable cancer. The surveys accompanying the vignette were completed by health services researchers knowledgeable on palliative care, practitioners, government officials, or teams consisting of a health systems expert working together with practitioners.

**Results:**

Completed vignettes were received from 8 countries: Bulgaria, Estonia, France, Lithuania, the Netherlands, Portugal, Sweden and England. Services provided for palliative care envisioned in the vignette’s pathway are, generally, covered by the statutory health systems. However, in some countries cost sharing exists for hospital stays, certain medicines and medical aids. Furthermore, coverage of social and financial assessments, home equipment and financial advice varied in nearly every country. Travel times to and availability of palliative care specialists were identified as challenges across nearly all countries. Organizational barriers, societal stigmas and knowledge gaps about what palliative care entails were also found to be areas in need of improvement.

**Conclusions:**

The comparative research presented provides further insight how countries organise palliative care, how services are offered and what levels of access exist around Europe. Our study showed differences in the scope of coverage of and access to the care options in the vignette. While responses showed countries have basic levels of coverage and access to services provided, there were variations, such as availability of specialists or the extent travel and waiting times influence care delivery. Settings where patients receive services also varied. As the need for palliative care grows in the future, health ministries and insurers should be increasingly concerned with how to guarantee coverage of and access to this care, as well as aware of best practices among countries facing similar challenges.

**Supplementary Information:**

The online version contains supplementary material available at 10.1186/s12904-025-01831-1.

## Introduction

Palliative care encompasses treatment and person-centred care for people with serious end-stage illnesses, such as incurable cancers. These cannot be cured or controlled with treatment and leads to death, and is also sometimes referred to as terminal cancer [1]. It primarily aims to improve and maintain quality of life for both patients and their families towards the end of the patient’s life. Secondary aims can include the consideration for the wellbeing of health and social care professionals. To achieve these aims, palliative care focuses on the assessment and management of pain and other symptoms between sectors of care, including a focus on care coordination, and necessitates an early identification and coordinated treatment strategy of problems in the physical, functional, psychological, social or spiritual dimension [2, 3]. Palliative care can be delivered in different settings depending on severity, life expectancy and the patients’ personal circumstances. These include private residences, assisted living facilities, rehabilitation, skilled and intermediate care facilities, acute and long-term care hospitals, clinics, hospice residences, correctional facilities, and homeless shelters [4].

Palliative care is strongly associated with cancer patients [5, 6]. Cancer is the second leading cause of death globally, about one in six deaths is due to cancer [7]. Neoplasms accounted for approximately 15% or all deaths in 2007 globally and 8% of total Disability-adjusted life years (DALYs), increasing to over 18% of global deaths and 10% of total DALYs in 2019 (most recent year), according to the Global Burden of Disease Study [8]. Patients with cardiovascular diseases, chronic respiratory diseases and neurological diseases frequently require palliative care as well.

According to the World Health Organization [9], palliative cancer care can encompass:

- Palliative cancer therapies: interventions that are intended to prolong life, such as chemotherapy, immunotherapy or radiation, accompanied by pharmacologic interventions to relieve suffering and assist quality of life leading up to death (neither postponing nor expediting)
- Supportive care: measures for the prevention and treatment of the consequences of cancer and its treatment over the course of the illness (e.g. management of physical, psychological and spiritual aspects of patient care)
- Respite care: addresses carers and enables them to recover from the strain of caring for severely ill patients (e.g. short-term care, substitute care or replacement care facilities; there may also be counselling for dealing with death and dying and family distress).

While there are detailed guidelines on cancer care available from many national and international associations [10–12], general guidance on palliative care for incurable illnesses remains a challenge given the intersectoral and coordination needs [13–15]. A main distinction of palliative care, however, is the emphasis on providing emotional and spiritual support in line with the patient’s goals and values, in addition to medical therapies [16].

Identified barriers to accessing palliative care are related to several factors, from the micro to the macro, including lack of knowledge surrounding palliative care (including not knowing what resources exist or knowing what palliative care actually is); patient preferences; health professionals’ palliative care knowledge, training and attitudes regarding end-of-life care; staffing and resources of palliative care units and hospices, particularly outside of urban settings; and integrating palliative care into the wider health and social care system(s) [17–21].

Palliative care has been receiving increasing socio-political attention in recent years, due to epidemiological developments and demographic change. Given its characteristics and the challenges described above, this paper compares access to palliative care services for adult patients with incurable cancer (given its strong association with palliative care) in eight European countries. It uses a vignette approach to identify differences in how palliative care services are covered, the different ways services are organized and provided, and the types of barriers patients might face in accessing them, hoping to provide sufficient granularity to support impetus for future action.

This research builds on work carried out by the European Observatory on Health Systems and Policies for the Expert Group on Health System Performance Assessment (HSPA) of the European Commission [22], aiming to explore the usefulness of the patient vignette approach as a complementary tool for identifying gaps and challenges in access to health care in the context of HSPA [23].

## Methods

### Conceptual framework

A vignette is a short description of a person or situation designed to simulate key features of a real-world scenario [24, 25]. These short descriptions are developed with expert input to create a hypothetical patient and characteristics, such as age, gender, medical complications and medical history. Completed vignettes are then usually presented to relevant professionals to solicit hypothetical responses, experiences or behaviours.

In medical literature, vignettes are mostly used to study variations in decision-making processes, including clinical judgments made by health professionals [26, 27]. The vignette methodology has been used in recent years to compare price levels in hospitals [28–30], gather insights on the availability and nature of outpatient medical care [31] and community dementia care [32]. In line with the goals of this work, the conceptual framework for using vignettes in identifying access barriers adopted by Palm et al. [22] was first used in other areas of care [see [33]]. We have adapted it to capture differences in coverage and access to services for the needs of a palliative care patient with incurable (lung) cancer.

### Palliative care vignette, survey design and participant selection

This palliative care vignette was based on available guidance for best practices of palliative cancer care, namely version 2.1 of the German guidelines (*S3-Leitlinie*) on palliative cancer care [34] and the Clinical Practice Guidelines of the National Consensus Project for Quality Palliative Care in the US from 2018 [4] that promote evidence-based practices for high-quality, safe and reliable provision of palliative care for patients with serious illness in various care settings; it was then reviewed by clinical oncology and palliative care experts and updated accordingly. The vignette details the diagnosis of the patient, their symptoms and hypothetical care plan and settings (Table 1) with the goal of studying variations across the eight countries surveyed in where patients are treated, how services are covered and how accessible they are. To maintain the generalizability of the vignette, we focused on the palliative care element of the patient pathway and not rapidly evolving treatment options, like immunotherapies.

**Table 1:** Palliative care vignette: patient description and services in the care pathway

Palliative care for cancer	Service
A 60-year-old patient with stage IV non-small cell lung cancer (5-year survival rate: 6%) has moderate to severe pain, breathlessness and acute moderate depression The oncology and specialist palliative care teams develop the following plan, including: - **Chemotherapy** to shrink tumours and alleviate symptoms - Opioids to treat background cancer **pain** and breakthrough pain as well as **breathlessness** and benzodiazepines to further control breathlessness and **anxiety** - Laxatives to prevent and aid with **constipation** due to opioid use (if the patient experiences severe constipation, an enema may be required) - **Psychological** assessment, followed by psychological support and/or antidepressants - **Advance care planning conversations** (regarding wishes for future care, for example cardiopulmonary resuscitation and preferred place of death)^a^ - **Carer assessment** followed by psychological support and bereavement support	*Oncology team*
	*Specialist palliative care team*
	*Chemotherapy*
	*Opioids:* - *Oral* - *Transdermal* - *Transmucosal* - *Injectable* *Benzodiazepines:* - *Oral* - *Injectable*
	*Laxatives* *Enemas*
	*Psychological therapies* *Antidepressants*
	*Social and financial assessment* *Home equipment* *Financial advice on how to access state financial support if needed*
	Advance care planning conversations
	*Services for carers* - *Psychological support* - *Respite care (patient admitted to an inpatient facility for a pre-determined period of time)* - *Bereavement support*

^a^These decisions should be recorded; with the patient’s consent, these decisions are recorded so this information can be shared between health care professionals

For the ease of collecting and summarising the data, a survey table was inserted next to the vignette’s listed services to capture the different dimensions of coverage of and access to the services in the vignette (see supplementary Table 1).

For individual countries, the respondents were either health services researchers knowledgeable on palliative care, practitioners, government officials, or teams consisting of a health systems expert working together with practitioners and other officials. As this is an exploratory study, there was no standard method or number per country. Rather, our country respondents ensured that at least one person with direct knowledge and insight into the care process was involved in filling out the survey. Respondents (hereafter experts) were first prompted to review and describe the care pathway in the vignette as regards population coverage, service coverage (focusing on which benefits are covered by the statutory health system) and cost coverage (what proportion of the cost is covered and what remains as an out-of-pocket (OOP) cost) in their country (Table 2).

**Table 2:** Coverage and financial protection

Coverage	
**Is the service covered by the statutory system?** (including exemptions)	**Does cost-sharing** (value or rule for determining the amount) apply? Any **financial protection** measures (e.g. lower cost-sharing for low-income groups/chronic patients, annual cost-sharing caps etc.)?

Experts were then asked to identify known or potential factors that influence gaps in accessing services in the survey (Table 3). These included [i] a lack of physical availability of services due to long distances, lack of statutory/contracted providers, poor quality of services, limited opening hours, waiting times and waiting lists; [ii] the patient’s potential (lack of) ability to obtain necessary care, such as the patient having an incapacity to formulate, obtain the care or to apply for coverage and fulfil the necessary requirements due to their condition or situation (e.g. people with cognitive impairment, mentally ill or homeless); [iii] any challenges or problems due to the attitude of the providers, for example due to discrimination (on age, gender, race, religious beliefs, sexual orientation, etc.) leading to care denial or inability to accommodate care to the patient’s preferences; and [iv] any other determinants that would worsen or improve access based on the vignette (e.g. determinants of age, sex, and socioeconomic status, insurance status, legal status, place of residence, as well as night vs. day treatment protocols, etc.).

**Table 3:** Physical availability and other determinants

Access			Determinants of access
**Is there a lack of *physical availability* of services** (e.g. due to distance, lack of statutory/contracted providers, poor quality of services, limited opening hours, waiting times and waiting lists)	Do patients lack the ***ability to obtain* necessary care?** (e.g. incapacity to formulate care request)	Do patients face problems due to the **attitude of the provider?** (discrimination, care denial, inability to accommodate preferences)	**Can you think of any factors that would worsen/improve access of this particular vignette? (**e.g. age, sex, and socioeconomic status, insurance status, legal status, place of residence, night/day, or anything else

We also asked experts to explain how the organization and provision of palliative care in their country differs from the vignette (Table 4) to provide further context on country-level experiences and contribute to the interpretation of other results.

**Table 4:** Country-level context

Actual care delivery for terminal cancer patients if different from the example above
If the above care plan differs from official recommendations and/or usual practice in your country, please comment on the differences in terms of services and treatments
Please comment on the setting the patient is most likely to receive these services in your country

Finally, we included additional space on the survey for experts (and encouraged them) to detail differences in the organization and provision of palliative care in their countries and include any relevant sources about coverage and access to these services.

### Sample, data collection and synthesis

The survey was sent to experts in eight countries: Bulgaria, Estonia, France, Lithuania, the Netherlands, Portugal, Sweden and the United Kingdom (England). The country selection was intended to include health systems with different operative features (i.e., social health insurance vs. tax-financed, multi- vs single payer, centralized vs decentralized) as well as geographical distribution within Europe.

**Fig. 1:**
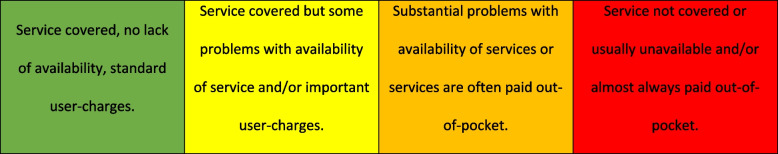
Colour coding guidelines to assess the survey responses

The survey responses were summarized in an Excel spreadsheet with one column per country to provide a side-by-side overview of the coverage of and access to the services in the hypothetical care plan and settings, with particular focus on the dimensions of coverage, cost-sharing and financial protection, and physician availability plus any other factors regarding barriers to care (see supplementary Table 2). Summaries for each of these domains per country were then captured on a single Excel spreadsheet and a colour coding scheme (green – yellow – orange – red) was used to visually compare results across the countries (Fig. 1). When more detail was necessary to understand the situation in participating countries, we followed up via e-mail.

The initial vignette surveys were sent out between June and October 2021; responses were received from the eight participating countries between July 2021 and June 2022. When additional information was needed, experts were contacted to provide further clarification (through April 2023).

## Results

The following sections detail the results on coverage, access to services, and relevant country context.

### Coverage of services

Services provided by the two care teams that are envisioned with developing the care plan (Table 1) in the vignette are, in general, covered by the respective statutory health systems. Where survey responses indicated that cost-sharing applied to services provided by an *oncology team,* this was related to potential associated hospital stays and were found in the Netherlands (where a mandatory deductible of EUR 385 per year applies to all medical care, except GP and home care, before insurance takes over), France (where some supplementary services, like deductibles for hospital accommodation, are only covered by voluntary health insurance (VHI)) and Sweden (where patients pay a fee of EUR 10 per day of hospitalization); these are not particular to palliative are, nor is such care exempt from them.

The situation across the surveyed countries varies more for how *specialist palliative care teams* are organized and covered, for example in the health systems of England, Estonia and France. Some services in England are provided by third sector hospice services, rather than the National Health Service (NHS) directly. The case in Estonia is that this service is not systematically defined, so as such it is not something that the Estonian Health Insurance Fund (EHIF) buys from service providers and rather the care and services encompassing palliative care are provided separately. Instead, the costs for psychologists, social workers and care coordinators (for example) are done on a fee-for-service basis, though the survey response indicates that changes to this piecemeal approach are under development. In France, patients are fully covered for medical care via the long-term conditions benefit (*Affection de Longue Durée*, ALD).

Survey responses indicated full coverage of chemotherapy treatments, with the only areas for incurred cost-sharing being the same as mentioned above for Sweden and the Netherlands. In Estonia, a financial instrument is used for new treatment schemes that shifts the burden of the cost between the EHIF and pharmaceutical companies, depending on the outcome of the treatment on the patient, i.e. the EHIF covers the costs when the patient’s situation improves, whereas the pharmaceutical company assumes the costs when not. Moreover, Estonia also has financial protection measures in place to guarantee EHIF coverage for the uninsured, who must apply for and be assigned disability status from a body called the Social Insurance Board or otherwise face full OOP payments. This is also seen in France, where persons qualifying for insurance schemes for low-income groups (*Complémentaire santé solidaire*, C2S) face no OOPs, whereas others in France would need VHI to avoid these. In the Netherlands, a monthly care allowance exists to help patients with lower incomes cover part of the annual deductible described above, though the patient may still incur OOP costs when seeking care from a provider that does not have a contract with their insurer.

Responses for the prescription of opioids and benzodiazepines, as well as psychological therapies and antidepressants, indicate good levels of coverage. Notable details were the existence of retail co-payments in Lithuania (with exceptions for those aged 75 +, disabled or retired and receiving low income, or those already having paid a certain amount of copayments), Portugal (with the state covering up to 90% of the cost) and Bulgaria, where benzodiazepines for outpatient treatment are not covered by insurance. Additionally, the most commonly used antidepressants in Estonia are covered with 50% co-payment and psychological support is not covered by the statutory health system in the Netherlands, unless provided within a GP practice or a mental disorder has been diagnosed (standard insurance does not cover adjustment disorders, such as coping problems of living with cancer).

Cost coverage for laxatives and enemas, on the other hand, are fully borne by patients in Portugal, with no indication of financial protection measures reported. The mandatory deductible (at this point reached by the chemotherapy treatments, according to the survey response) also applies to physician prescribed laxatives and enemas in the Netherlands and reimbursement varies in Estonia (for example Naloxegol is covered with 75% co-payment for long-term (2 months) opioid-induced constipation). Survey responses for other countries indicate full cost coverage for laxatives and enemas, for example there are two laxatives that are fully covered in hospital care in Bulgaria.

The extent to which social and financial assessments, home equipment and financial advice are covered varied in nearly every country surveyed. Regarding coverage of home equipment, there is means testing for potential benefits for patients (England) as well as new coverage via approval of a physicians’ commission (Bulgaria). In Estonia, the costs for social workers in health care institutions are covered by the EHIF. Home equipment is not regularly financed but can be organized by local government. In the Netherlands, municipalities, trade unions (for their members) and social care teams are involved in providing financial advice on accessing state aid, with nurses referring patients to the municipalities responsible for running the social care scheme.

While Dutch insurers are responsible for providing equipment in the short term (up to six months), this becomes the job of municipalities after six months (for prolonged use, i.e. hoists, shower chairs, wheelchairs), and may implicate that patients have to re-apply at another window when care becomes prolonged. For equipment provided via the municipality, a small deductible is charged (EUR 19 per month, except for wheelchairs). In Lithuania, home equipment is covered by the National Health Insurance Fund and social workers, who are part of the multidisciplinary palliative care team, may assist patients to obtain aid from their municipality.

Social care (particularly an issue for older patients) was reported not always well covered in France, though VHI and cancer associations are used to improve coverage.

Expert responses on the coverage for advance care planning conversations also show differences. They were reported to be fully covered (without cost-sharing) as part of routine care in Bulgaria, England, the Netherlands and Portugal and non-existent/non-routine in Estonia and Sweden. Survey responses from France and Lithuania noted that information for this was not available.

Finally, coverage of services for carers (psychological support, respite care and bereavement support) were covered via statutory health insurance, the social care system, local governments or external organizations (England, Estonia, Lithuania, Sweden, Portugal), or only partially covered by these different entities with some cost-sharing (Bulgaria, France, the Netherlands).

### Access to services

Our survey prompted experts to identify known factors that influence barriers to accessing services for the different care points in the vignette (Table 3). For seeing *oncology teams*, *specialist palliative care teams* and receiving chemotherapy services, travel times were identified as problematic across all countries surveyed, except for the Netherlands, and survey responses focused on the fact that carers and care facilities are concentrated in larger cities, requiring rural and suburban patients to travel to their regional or national capital, provided they have reliable transportation or are physically able to travel. This poses additional barriers for low-income patients.

Waiting times for care were also reported as problematic in England and Estonia, with waiting time targets established for oncology services being missed in the latter two. For Estonia, the lack of personnel is a particular concern, as there are only three specialist palliative care centres in the country and their overall effectiveness/added value remains unknown due to a lack of evaluation. In Lithuania, physicians who complete a minimum of 44 h of training are eligible to work in multidisciplinary teams^1^ to provide inpatient or outpatient palliative care, though a large challenge is the lack of physicians. This is attributed to risks associated with uncertainties associated with ambiguous regulations (e.g. when determining whether a patient's condition meets the criteria for prescribing palliative care or suitable for nursing) and inadequate financing (e.g., low tariffs, unpaid remote consultations, etc.).

Travel times may also play a role in less densely populated countries where existing infrastructure for palliative care can differ: only about half of Sweden’s 21 regions have specialist palliative care hospital wards, for example, with 12 hospices suitable for palliative care patients across the country. The need for more inpatient infrastructure and home care teams was also described in Portugal, and practitioner-patient approaches to palliative care were identified as hindering access to in several countries (see below).

Regarding barriers in accessing opioids and benzodiazepines, laxatives and enemas, and psychological therapies and antidepressants, the survey responses identified three areas: organizational barriers, societal stigmas and knowledge gaps. Organizational barriers in this context primarily refer to either limited pharmacy opening hours and distances, as identified in Bulgaria and England, or with personnel, as in Estonia, France (lack of psychological professionals in some areas of the country or in some facilities) and Lithuania (restricted access to psychiatrists responsible for routinely prescribing initial medications, even when family physicians may reissue them for a period of time). Stigmas and feelings of shame or guilt, or simply the patient’s fear of these things was identified in the responses as challenges regarding drugs and psychological therapies in England, Estonia, Portugal and Sweden. Finally, the sensitive nature of these treatments highlighted the need to facilitate full explanations and provide ongoing support on the benefits were identified in England. On the other hand, though, experts in both Estonia and Sweden attributed the knowledge gaps creating barriers to access drugs and psychological therapies first and foremost to lack of experience in prescribing them as well as a general fear of prescribing them, particularly opioids.

Pertaining to access of services of social and financial assessments, home equipment, financial advice, advance care planning conversations, and services for carers in the vignette, country responses further illustrate the cross-sectoral and integrated nature of palliative care. For instance, areas in England with low densities of social workers and benefits advisors result in less uptake of these services in the statutory system, even when patients and their families are well-informed about what support could or should be available. In Estonia, the issue is a lack of systematic and regulated cooperation between the health and social systems. No system for advance care planning conversations currently exists in Estonia, while in France wide variations can be observed, indicating a lack of standard practices. The provision of limited respite care services in Lithuania is predominantly reliant on the social system and patients reportedly benefit from the assistance of social workers on the multidisciplinary palliative care team and connect them with municipal social services and allowances. Social workers were also described as being scarce within local communities and mainly confined to hospitals in Sweden, while the carers may be unaware of the existing possibilities to get support for themselves.

### Country-level context

While our vignette lays out a care plan developed by oncology and specialist palliative care teams, this did not necessarily reflect reality for all participating countries. For example, care in Lithuania is spearheaded by a general physician (GP) and mostly provided by nurses within multidisciplinary team arrangements. Additionally, the use of benzodiazepines to treat breathlessness and anxiety is not part of palliative care planning in Bulgaria, while psychological assessments in the country are only available at some of the comprehensive oncology centres (COCs) and oncology hospital wards, which are responsible for inpatient palliative care in Bulgaria, and carer assessments/psychological support are not included. The French National Authority for Health (*Haute Autorité de Santé*), on the other hand, only mentions support for grieving families and close relatives in its recommendations for the organization of palliative care, though not explicitly for carers tasked with supporting the patient [35].

## Discussion

Examining palliative care for a patient with incurable cancer in eight European countries yielded differences in both the scope of coverage of and access to the numerous care options laid out in the vignette. While all survey responses indicated at least basic levels of coverage and access to services provided by oncology teams, specialist palliative care teams, chemotherapy treatment and select drugs (opioids and benzodiazepines), there were variations seen, such as the availability of specialist palliative care teams or the extent to which travel times and waiting times influence care delivery. As travel times are an access barrier in most of the countries surveyed and that patients outside of urban areas may struggle for physical or financial reasons to travel, France and England provide examples of how to overcome these geographic disparities with mobile palliative care teams [36, 37].

Furthermore, the settings where patients are most likely to receive health services also showed variety. In England, the distribution of care between outpatient and inpatient settings matched very closely with the guidelines in Germany that partially inspired the vignette, suggesting a convergence in palliative care in larger health systems [34]. In Sweden, patients with palliative care needs are likely to end up in different hospital wards (internal medicine, general surgery, gynecology) instead of in a general or oncology ward as outlined in the vignette. Specialist palliative outpatient clinics and day hospice care are also very rare. Hospice care in Estonia was reported to be limited, with only three across the country (two in Tallinn and one in Tartu), with a total of 40 beds for patients. Finally, regional expert teams have been created in the Netherlands to advise health professionals concerning the care of specific patients in cases where specialist palliative care teams (which exist in hospitals, home care and nursing homes in the country) are not available.

Additionally, the existence of barriers stemming from the practitioner-patient relationship was detailed in five of the eight countries surveyed. For Bulgaria and France, this meant that there are concerns about physicians’ abilities to thoroughly explain palliative care to patients and assuage any lingering concerns regarding the options for treatment. For Estonia, this involved specialist care teams not prioritising incurable patients, while a poorer quality of care was identified as a problem for certain minority groups in England. In Sweden, existing prejudices were reported among patients and health care staff alike about what palliative care is and if it is even a useful target of resources.

While our survey primarily focused on how health systems provide palliative care based on a hypothetical pathway, experts were also able to reflect on their country-level contexts, revealing substantial variation in palliative care between countries. The comparative research presented in this article thus provides further insight as to how countries organise palliative care, how services are offered and what levels of access exist around Europe. Understanding this is even more important with ageing populations and higher disease burdens, but also to account for how any limited service coverage and access impacts vulnerable and marginalized groups even more, particularly in the context of having capacity to lead a valued and meaningful life given their unique circumstances (also known as existential health) [38].

Finally, communication on palliative care (among and between practitioners, family members, carers and others, is a critical topic. Whether it be to overcome scepticism of its application or stigmas around some of its services, it is very likely to be part of further palliative care planning in and beyond the eight countries we surveyed. Given that palliative care sees services move from the clinical side with the “life-changing diagnosis” increasingly towards social services for all parties involved, the availability of and familiarity with palliative care on the whole thus relies on effective communication. For example, this is why improved communication was included as a key recommendation in the recently released strategy draft document on palliative care from the Government of Malta [2, 3, 39].

### Methodological Considerations

This study has some methodological limitations. First, a notable limitation of vignettes is that they may not accurately represent real-world scenarios, both in terms of textual descriptions and the hypothetical behaviours they elicit. This discrepancy can arise due to factors such as social desirability bias among respondents, potentially undermining the validity of the results and conclusions [27, 40]. As there is no “typical case” for palliative care and definitions may vary across countries, our developed vignette had to be broad, though this is why we included additional space on the survey for experts to detail differences in the organization and provision of palliative care in their countries. Given that some examined countries have more nationally relevant scientific literature or national guidelines on palliative care than others, information availability was not uniform across the sample. To minimize bias among respondents, we engaged health services researchers with expertise in palliative care, alongside practitioners, government officials, and collaborative teams consisting of health systems experts working in tandem with practitioners and other officials.

Second, given the differences in national standards for palliative care pathways, we decided to develop the vignette based on countries with recently released guidelines (Germany and the United States of America), with validation from palliative care experts; as these countries were not part of the study, this may have limited the applicability of the vignette. Third, the level of detail on the information in the survey responses did vary, though we followed up with clarifications to original responses from experts where necessary in order to conduct the cross-country analysis of the results. Fourth, while experts also consulted palliative care experts in their own countries to complete the survey, we provided the survey in the English language only, meaning terminologies could have been misinterpreted by experts. Finally, with rapid developments in the fields of personalized and precision medicine and developments in areas such as immunotherapy, our data originally collected in 2021–2022 may miss some more recent developments in the evolution of care. However, as our intention was to focus on the palliative care element of the patient pathway, and while this will certainly also evolve as a consequence of ongoing developments in immunotherapy, we are convinced that findings remain timely and should inform policy moving forward.

## Conclusion

As the need for palliative care grows in the future, health ministries and insurers will be increasingly more concerned with how to guarantee coverage of and access to this care, as well as how other countries go about this. This study provides insights into existing frameworks for care, clinical pathways and current challenges. Moving forward, countries will need to see if standardising their pathway with internationally agreed-upon guidelines simplifies or complicates things at the patient and carer levels, as changes at the national and regional levels undoubtedly have impact on the delivery of care. To better ensure that palliative care is truly patient-centred, counties should also support the co-design of palliative care patient pathways and services, and as well as communication between providers and payers. The emerging field of personalized medicine based on analysing an individual’s genetic traits further presents opportunities to design treatments catered specifically to one’s needs and can alter traditional service delivery methods, though the current high costs associated with this must be considered as well [41]. Additionally, ensuring workforce sustainability and resilience, promoting better coordination of care and transparency in the health system can all aid in avoiding hypothetical disruptions to care that were shown in the survey responses described above. Finally, as health systems undergo other changes in the coming years, it will be critical to see to what extent addressing other existing issues in health systems impact new and existing frameworks regarding palliative care.

## Supplementary Information

Supplementary Material 1.

Supplementary Material 2.

## Data Availability

No datasets were generated or analysed during the current study.

## Footnotes

1 includes physicians, nurses, nursing assistants, medical psychologists, physiotherapists, and social workers.
