# Supplementary material for: The variation of coverage and access to palliative care for cancer patients in eight European countries: an exploratory vignette approach
Source: BMC Palliat Care. 2025 Jul 7;24:188. doi: 10.1186/s12904-025-01831-1 (PMC12235821; doi:10.1186/s12904-025-01831-1)
Supplement: Supplementary file 1 — Supplementary Material 1. [file 12904_2025_1831_MOESM1_ESM.pdf]

Vignette

**Palliative care for cancer**		**Coverage**		**Access**			**Determinants of access**
A 60-year-old patient with stage IV non-small cell lung cancer (5-year survival rate: 6%) has moderate to severe pain, breathlessness and acute moderate depression.<br><br>The oncology and specialist palliative care teams develop the following plan. including:<br>- **Chemotherapy** to shrink tumours and alleviate symptoms<br>- Opioids to treat background cancer **pain** and breakthrough pain as well as **breathlessness** and benzodiazepines to further control breathlessness and **anxiety**<br>- Laxatives to prevent and aid with **constipation** due to opioid use (if the patient experiences severe constipation, an enema may be required)	**Service**	**Is the service covered by the statutory system?** (including exemptions)	**Does cost-sharing** (value or rule for determining the amount) apply? Any **financial protection** measures (e.g. lower cost-sharing for low-income groups/chronic patients, annual cost-sharing caps etc.)?	**Is there a lack of *physical availability* of services** (e.g. due to distance, lack of statutory/ contracted providers, poor quality of services, limited opening hours, waiting times and waiting lists)	Do patients lack the ***ability to obtain* necessary care?*** (e.g. incapacity to formulate care request)	Do patients face problems due to the **attitude of the provider?**** (discrimination, care denial, inability to accommodate preferences)	**Can you think of any factors that would worsen/improve access of this particular vignette? (**e.g. age, sex, and socioeconomic status, insurance status, legal status, place of residence, night/day, or ANYTHING else
	Oncology team						
	Specialist palliative care team						
	Chemotherapy						

- **Psychological** assessment, followed by psychological support and/or antidepressants<br>- **Advance care planning conversations** (regarding wishes for future care, for example cardiopulmonary resuscitation and preferred place of death)[1]<br>- **Carer assessment** followed by psychological support and bereavement support	*Opioids:*<br>- *Oral*<br>- *Transdermal*<br>- *Transmucosal*<br>- *Injectable*<br>*Benzodiazepines:*<br>- *Oral*<br>- *Injectable*						
	Laxatives<br><br>*Enemas*						
	Psychological therapies<br><br>*Antidepressants*						
	Social and financial assessment<br><br>*Home equipment*<br><br>*Financial advice on how to access state financial support if needed*						

[1] These decisions should be recorded; with the patient's consent, these decisions are recorded so this information can be shared between health care professionals

	Advance care planning conversations						
	Services for carers<br>- *Psychological support*<br>- *Respite care (patient admitted to an inpatient facility for a pre-determined period of time)*<br>- *Bereavement support*						

*due to a person's incapacity to formulate care request, obtain the care or to apply for coverage (and fulfil the necessary requirements) due to their condition or situation (e.g. people with cognitive impairment, mentally ill, homeless); ** for example due to discrimination (on age, gender, race, religious beliefs, sexual orientation, etc.) leading to care denial or inability to accommodate care to the patient's preferences.

Actual care delivery for terminal cancer patients if different from the example above

**If the above care plan differs from official recommendations and/or usual practice in your country, please comment on the differences in terms of services and treatments**	
**Please comment on the setting the patient is most likely to receive these services in your country (see example from Germany, below)**	

Possible pathways/setting for palliative care in Germany (Leitlinienprogramm Onkologie, 2020).

	Implementation of a generalist palliative care intervention (11.5.3)	Implementation of a specialist palliative care intervention (11.5.4)		
In- patient	General hospital ward/ oncology ward/ nursing home	Palliative care unit (11.5.4.1)	Hospital pallia-tive care sup-port team (11.5.4.2)	Palliative care day centre (11.5.4.5)
Out- patient	General out- patient palliative care	Specialist palliative home care (11.5.4.3)	Specialist palliative out-patient clinic (11.5.4.4)	Day hospice (11.5.4.5)
Overlapping sectors	In- patient hospice (11.5.6)			
	Hospice services/volunteer- based services (11.5.5)			
